# Supplementary figures and images for: High-Resolution Transcriptome Analysis with Long-Read RNA Sequencing
Source: PLoS One. 2014 Sep 24;9(9):e108095. doi: 10.1371/journal.pone.0108095 (PMC4176000; doi:10.1371/journal.pone.0108095)

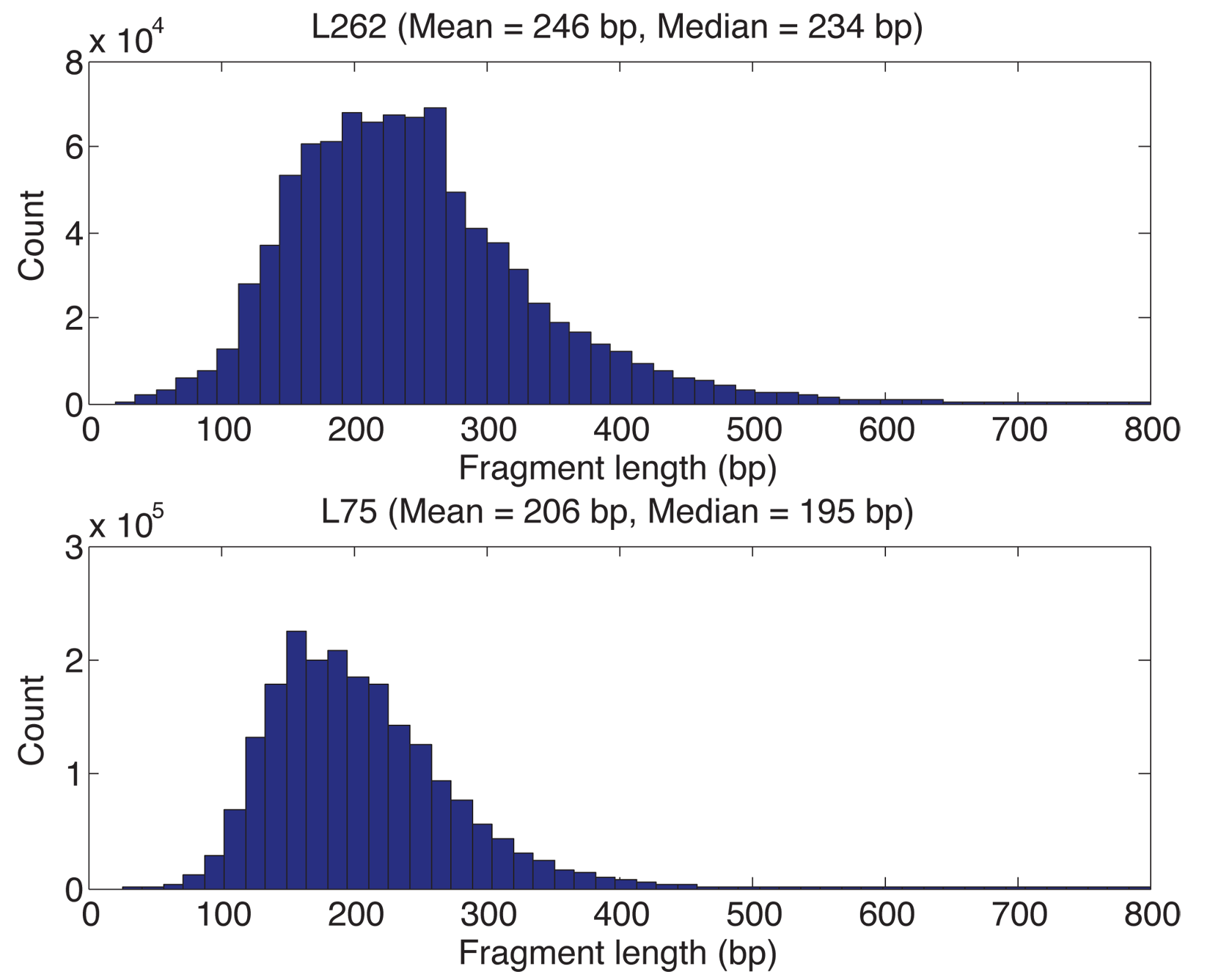

Supplement: Figure S1 — Estimated fragment length distribution. We used the reads that mapped to genes with exactly one transcript to estimate the fragment length distributions for L262 and L75. Fragment length was measured by counting the number of exonic nucleotides between the leftmost and rightmost bases (inclusive) to which a given read pair was aligned. The read alignments produced by GSNAP were used. Using our reduced fragmentation time for long-read library generation (1 min vs 8 mins), we did not observe striking shift in the fragment size distribution suggesting that increases in this distribution requires protocols complemented with a gel-based size selection after fragmentation. (TIF) [file pone.0108095.s001.tif]

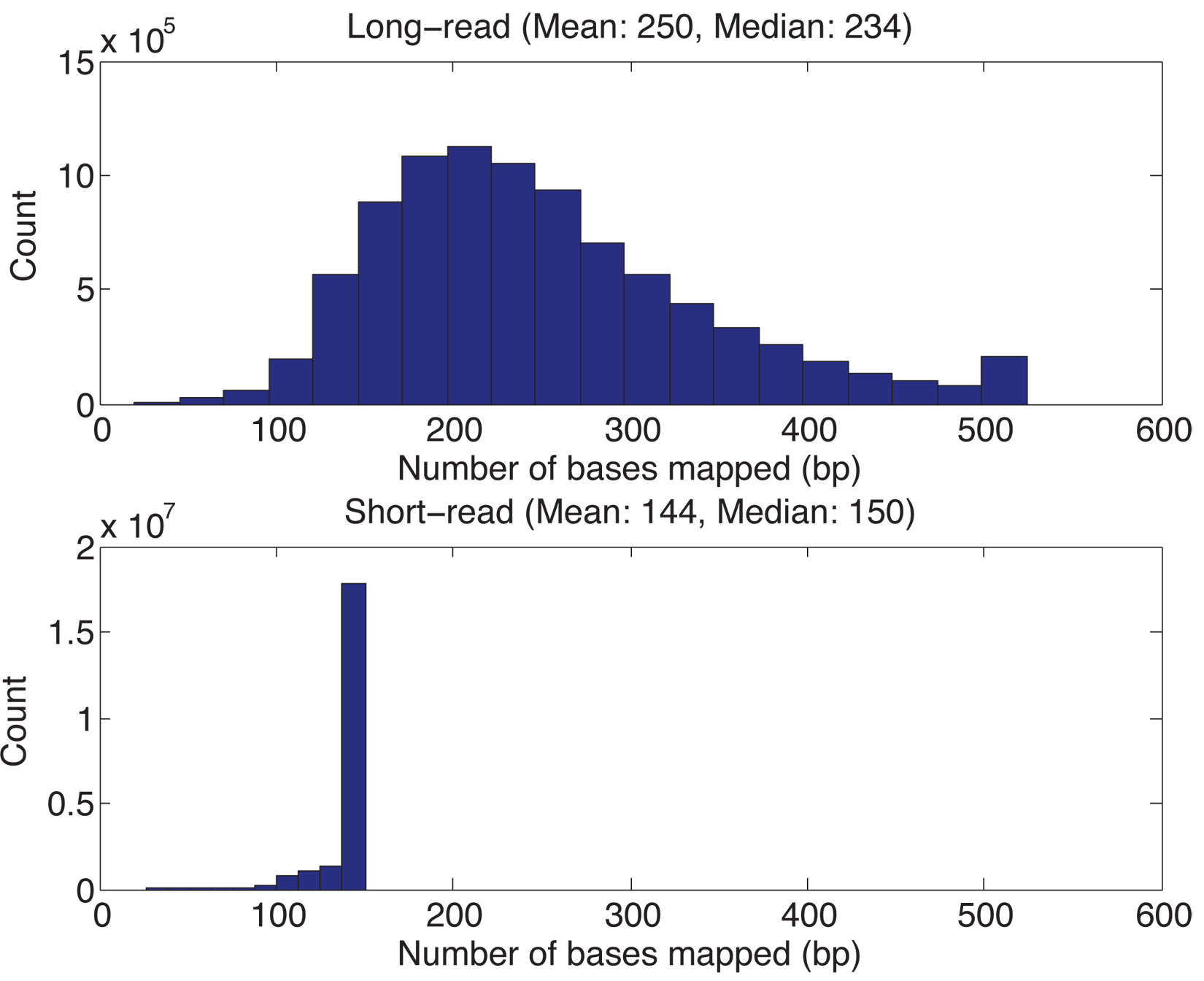

Supplement: Figure S2 — Histogram of number of reference bases covered by each read. As a measure of information content, we counted the number of bases in the reference genome covered by each read pair. For overlapping reads, for instance, this will be less than double the read length. From the histograms, we can see that the vast majority of reads in L75 are non-overlapping while reads in L262 show high degrees of overlap. Overall, however, L262 reads still have more information content than L75 reads. (TIF) [file pone.0108095.s002.tif]

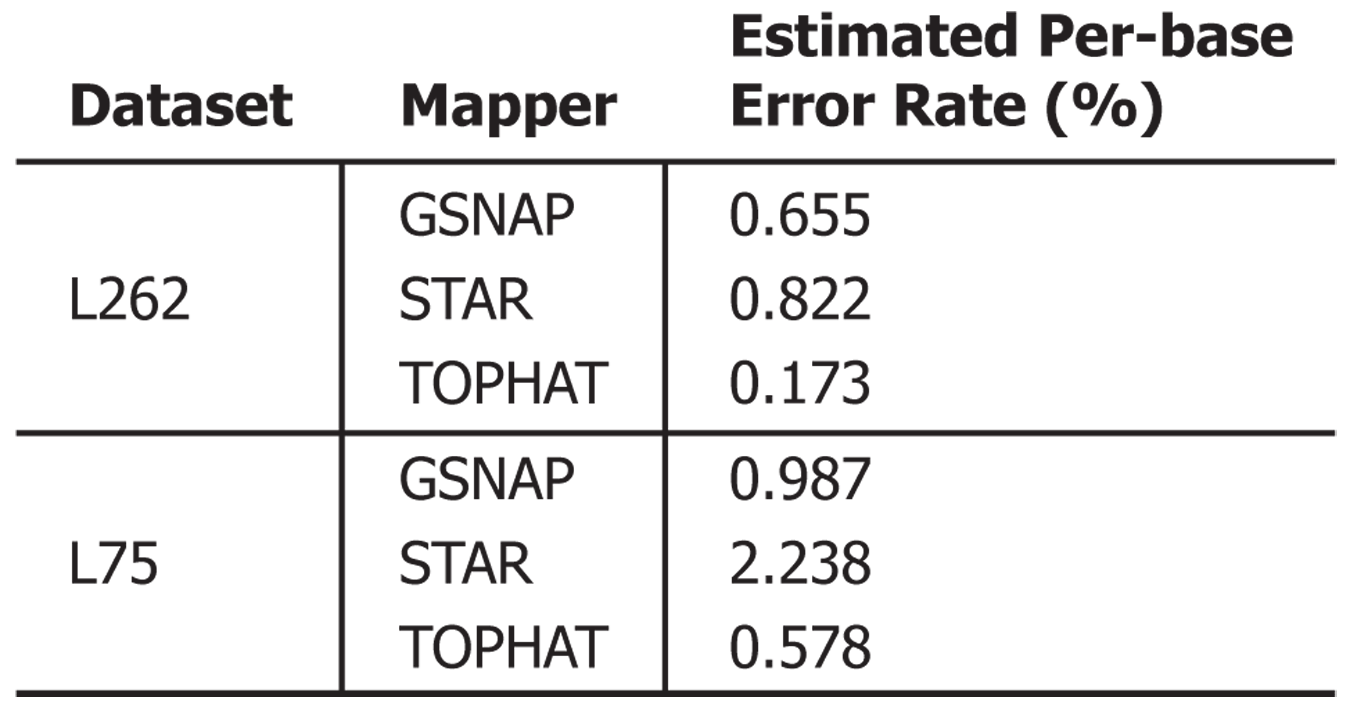

Supplement: Figure S3 — Estimated per-base sequencing error rate for each aligned dataset. Sequencing error rate is estimated as the total number of mismatches in the alignments divided by the total number of aligned bases. (TIF) [file pone.0108095.s003.tif]

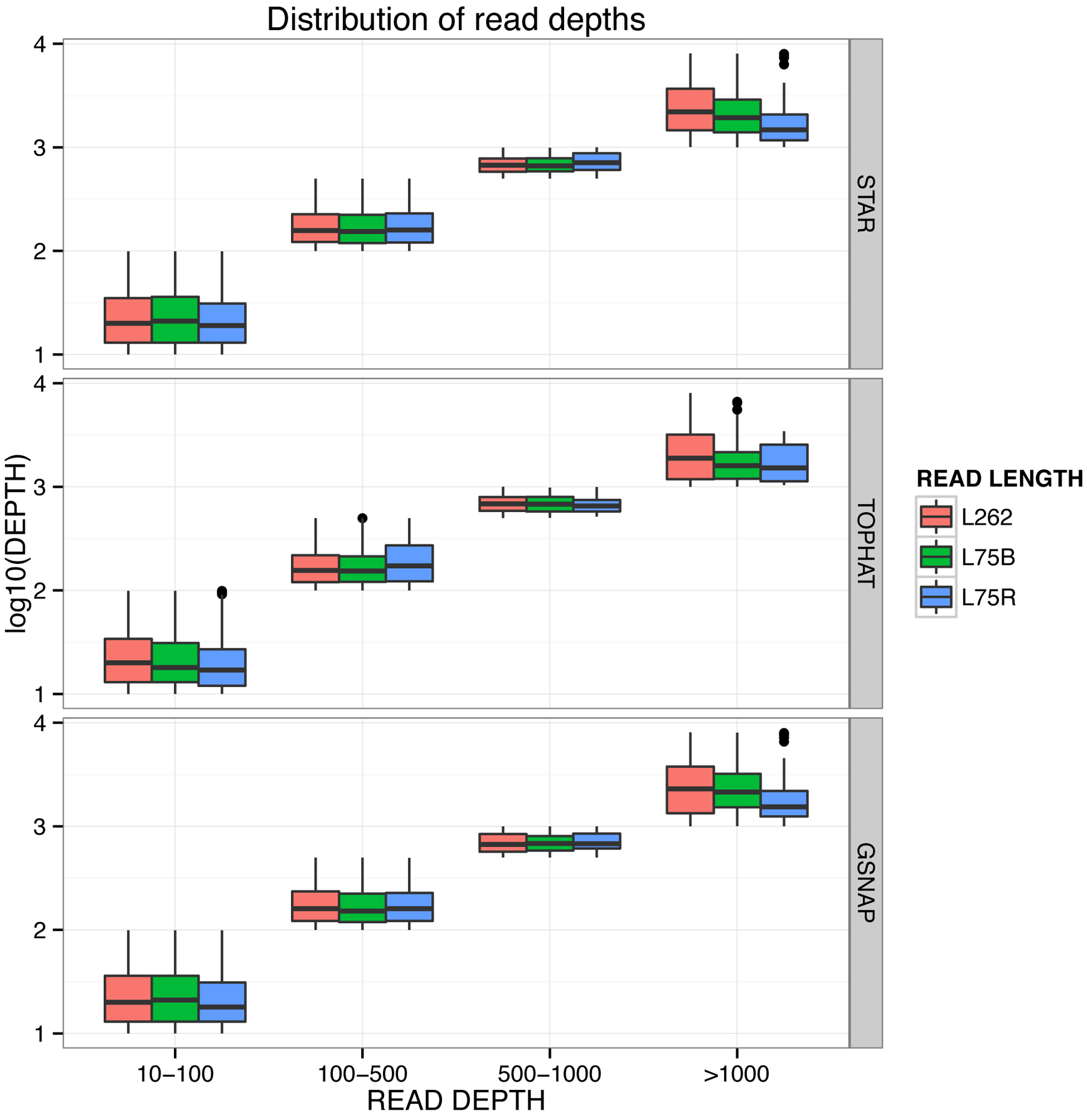

Supplement: Figure S4 — Comparison of read depth distributions. Genotyped sites were stratified into the same four read depth bins as used in the genotype concordance analysis. This plot compares the distribution of read depths within each category across mappers and read length. The distributions within each read depth category are highly similar across read lengths. L75 was randomly subsampled down to the same number of bases (L75B) and the same number of reads (L75R) as L262. (TIF) [file pone.0108095.s004.tif]

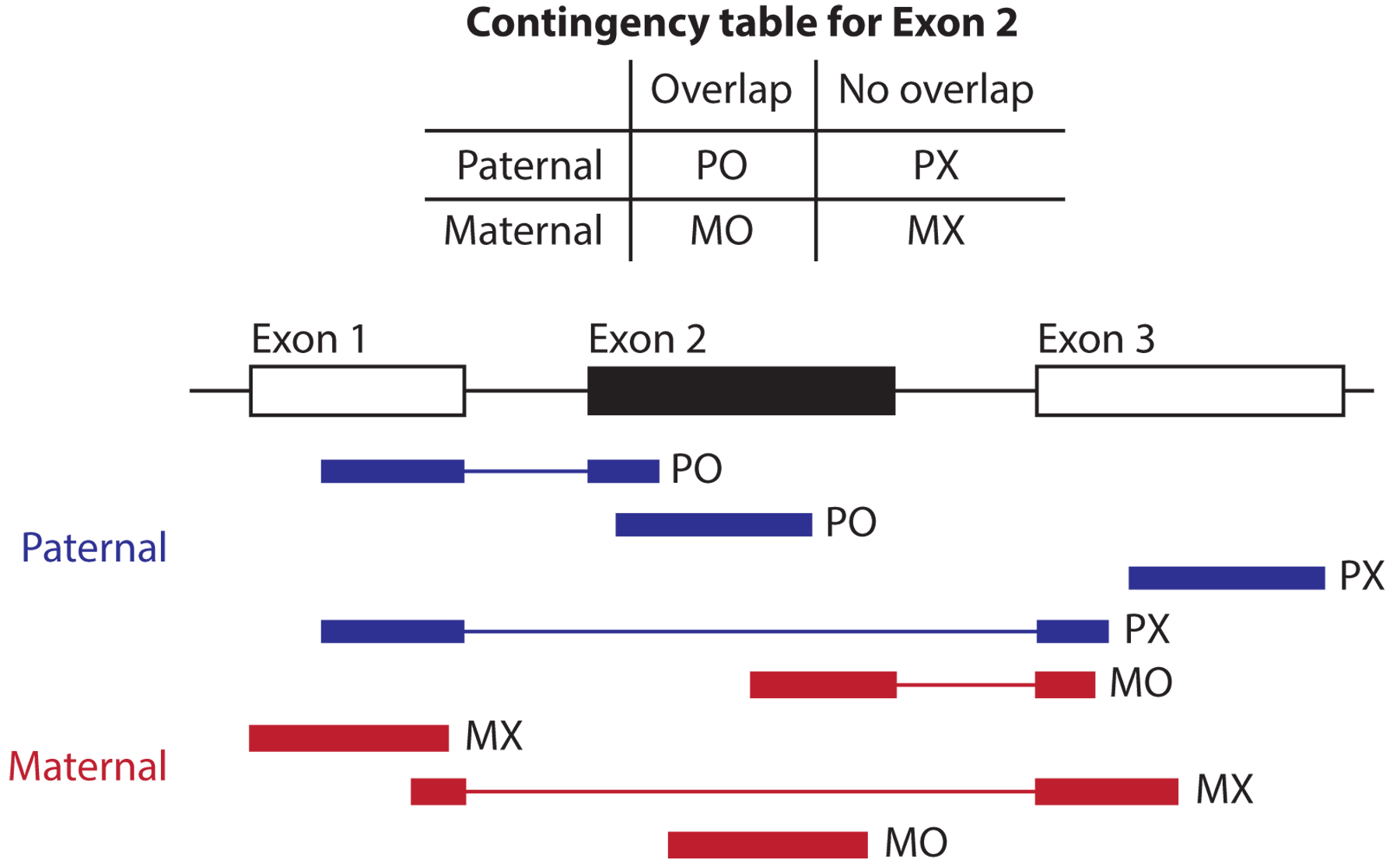

Supplement: Figure S5 — Illustration of differential exon usage test. We tested whether each exon block exhibits a differential usage pattern using a chi-square test on a 2-by-2 contingency table where the rows indicate whether a read is maternal or paternal and the columns indicate whether a read overlaps with the exon block of interest. Each example read (shown as single-end for simplicity) is marked with the cell in the contingency table it is counted towards. (TIF) [file pone.0108095.s005.tif]

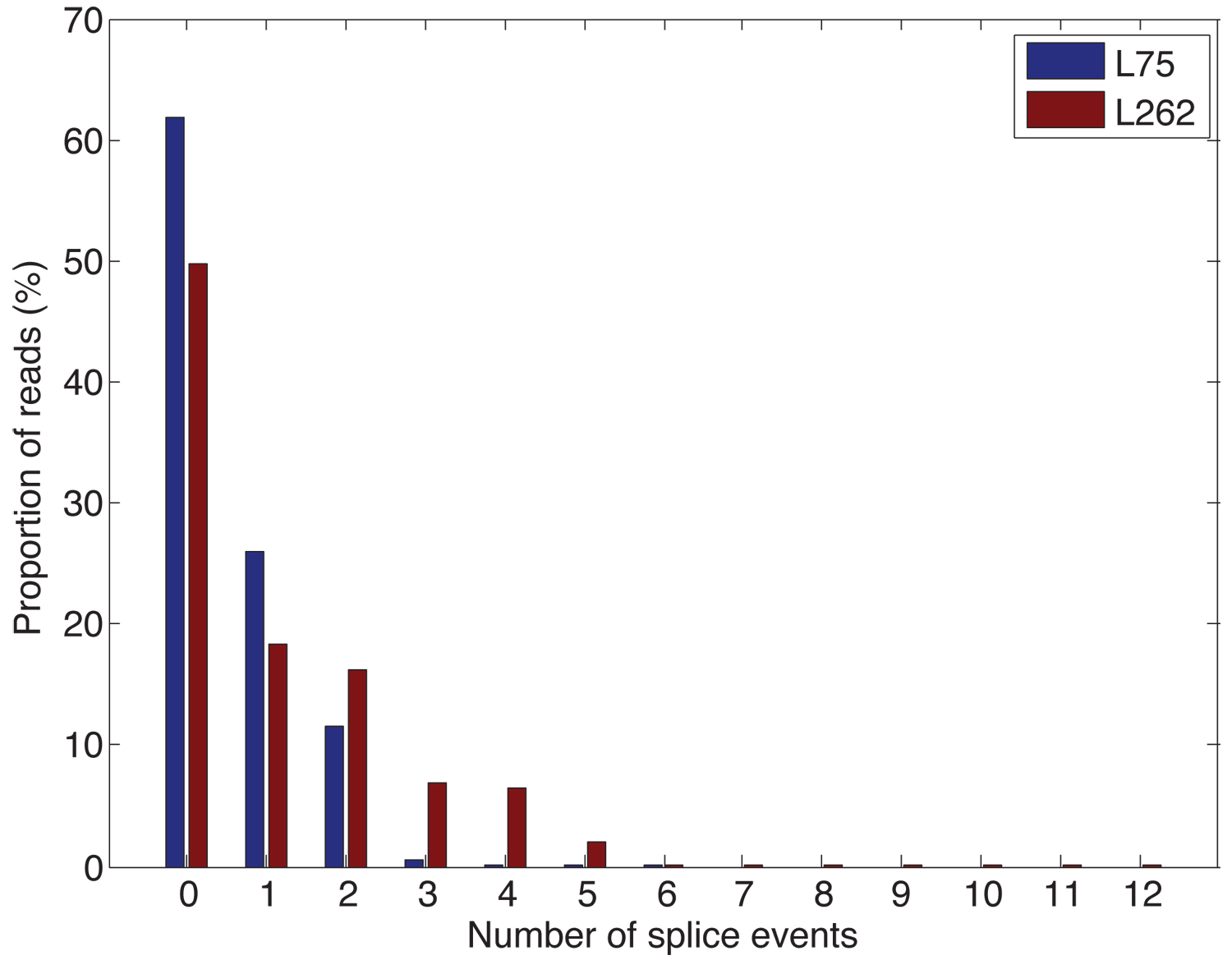

Supplement: Figure S6 — Distribution of number of splice junctions spanned by each read. For each read pair, we counted the number of splice events represented in the alignment from GSNAP. The bar graph shows the distribution of this number in both L262 and L75. We can see that L262 reads tend to span more splice junctions than L75. (TIF) [file pone.0108095.s006.tif]

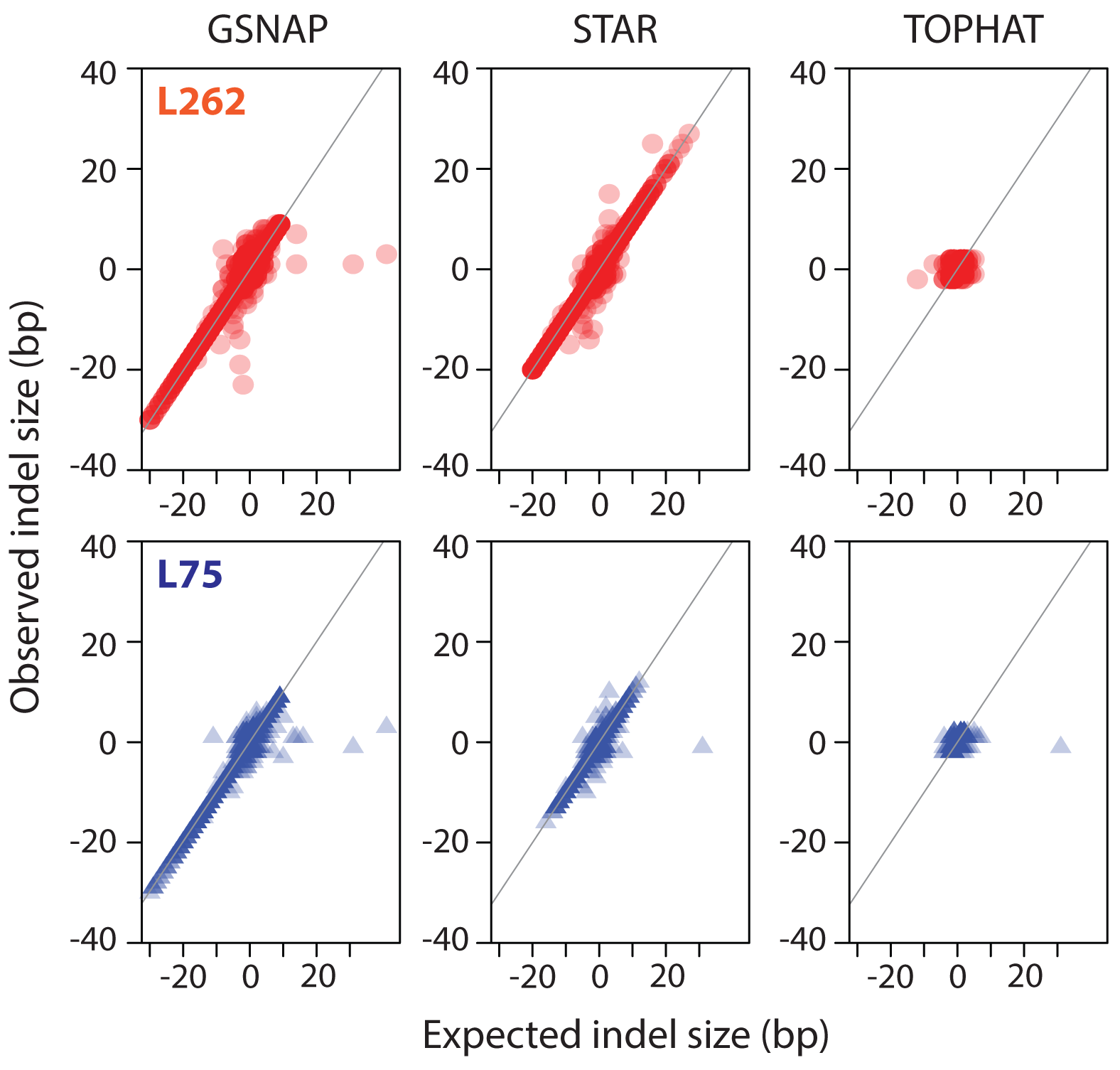

Supplement: Figure S7 — Expected indel size from DNA-seq compared to most frequently observed size from RNA-seq for L262 and L75. Points along the diagonal (gray line) in each plot indicate a positive correlation between most frequently observed indel size and expected size based on genotyping. In general, STAR and GSNAP perform reasonably well in mapping indels as large as 20 bp in length. Tophat, on the other hand, is very limited in its ability to call indels, maxing out in its ability to call an indel at 2 bp in length. STAR appears to perform equally well in mapping insertions and deletions, performing best with the long reads. GSNAP appears to have a bias in mapping deletions. The L75 dataset was subsampled to match the number of bases in L262 for this analysis. (TIF) [file pone.0108095.s007.tif]

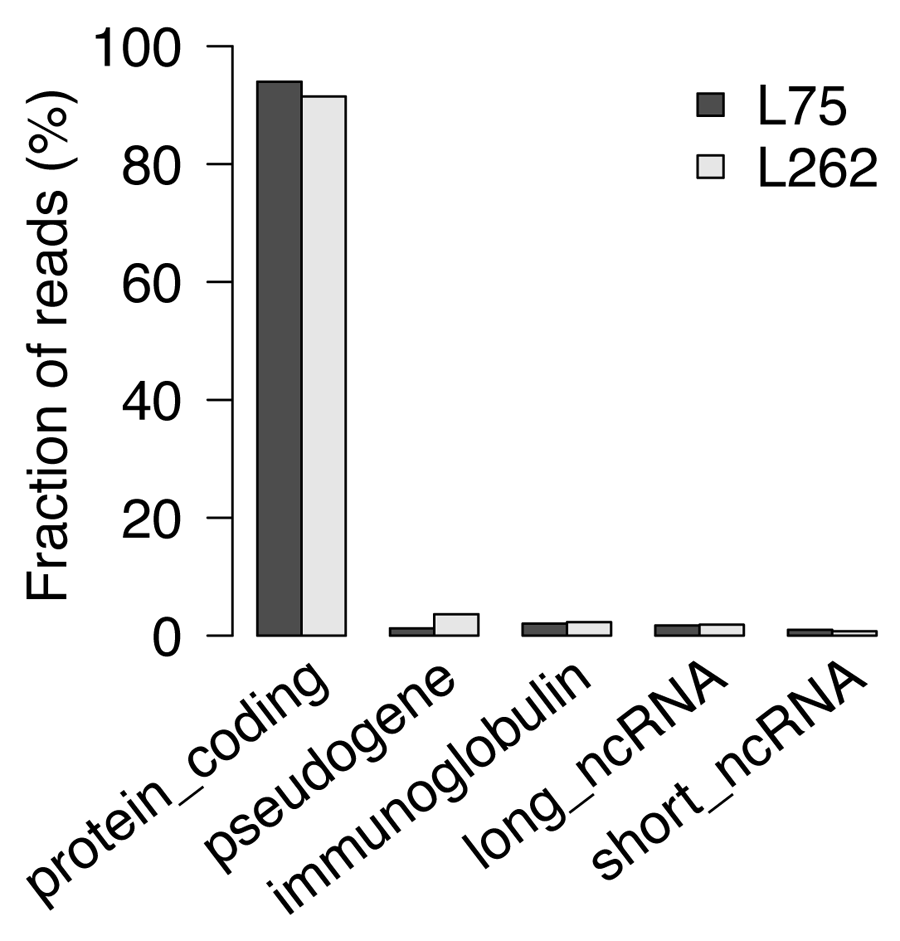

Supplement: Figure S8 — Breakdown of reads by gene category. The fraction of reads mapped to each of the five major gene categories in GENCODE annotation (version 15) is shown. L262 displayed a relatively high proportion of reads originating from pseudogenes compared to L75. (TIF) [file pone.0108095.s008.tif]

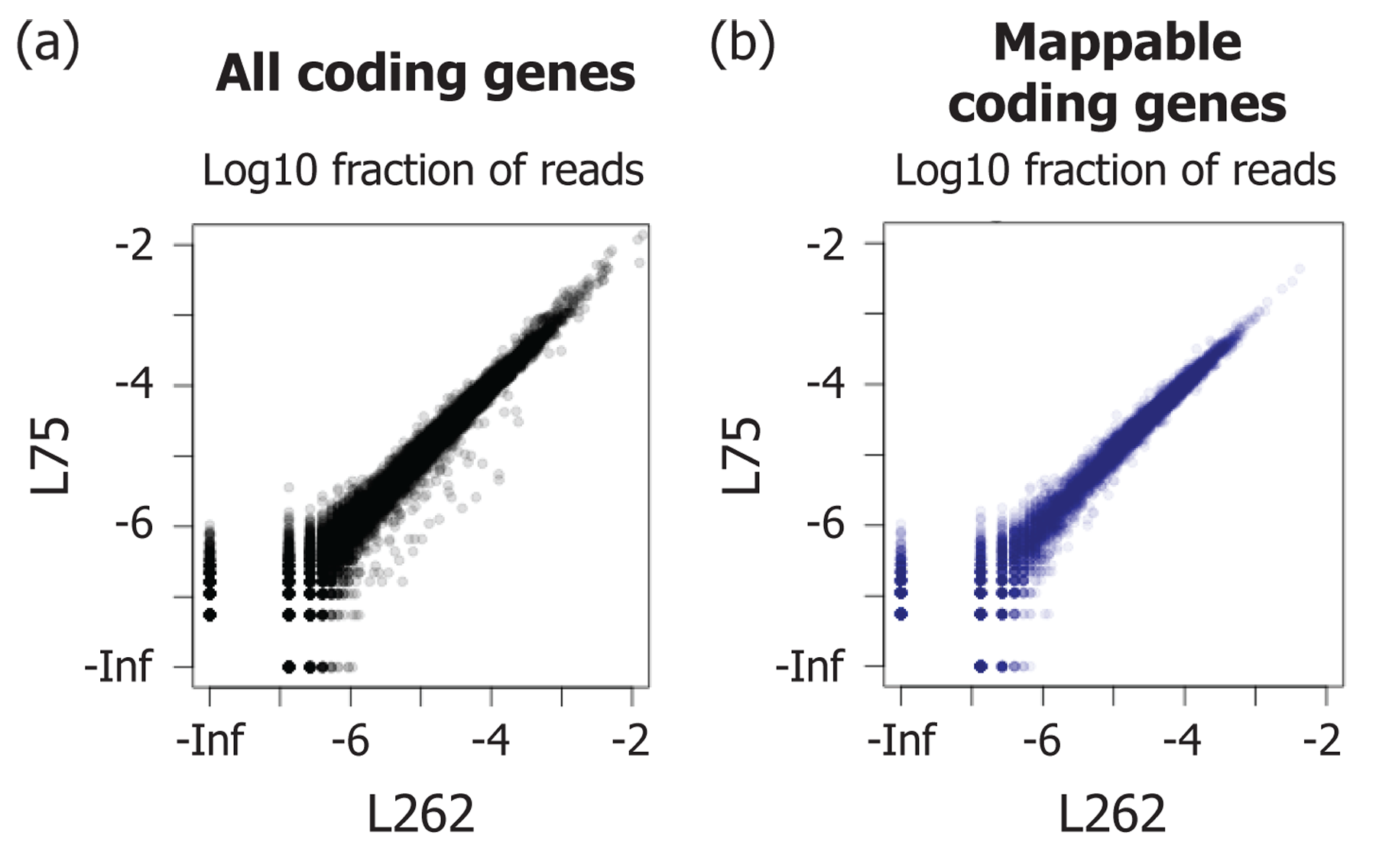

Supplement: Figure S9 — Comparison of gene quantification for protein coding genes. Log scatter plots of fraction of reads mapped to each gene between L262 and L75. In (b), only the genes with perfect mappability scores are plotted. (TIF) [file pone.0108095.s009.tif]

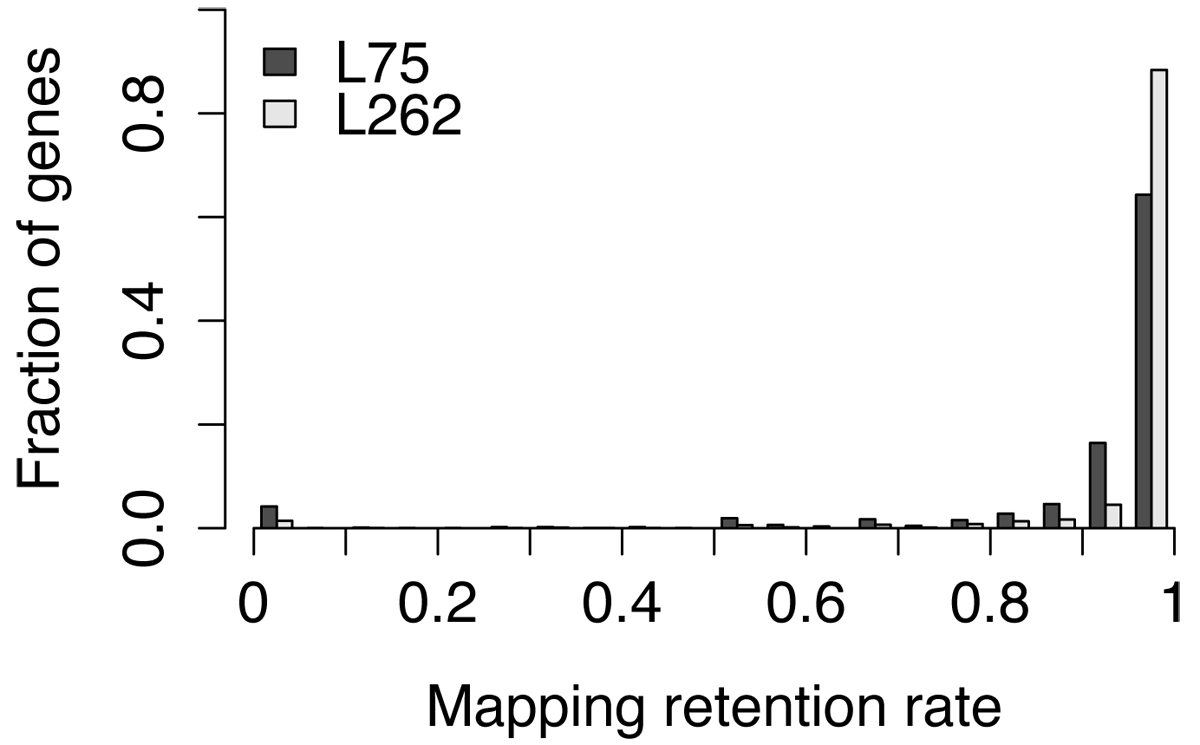

Supplement: Figure S10 — Genes quantified by longer reads display higher mapping retention rate. Mapping retention rate is a simulation-based metric that is inversely correlated with the severity of allelic bias in allele-specific quantification. It is computed for each gene as the proportion of reads that map to the same location after flipping all bases that align with heterozygous sites in the reference so that the new bases are the alleles from the other parental haplotype. The above histogram compares the distribution of mapping retention rates in L75 versus L262. (TIF) [file pone.0108095.s010.tif]

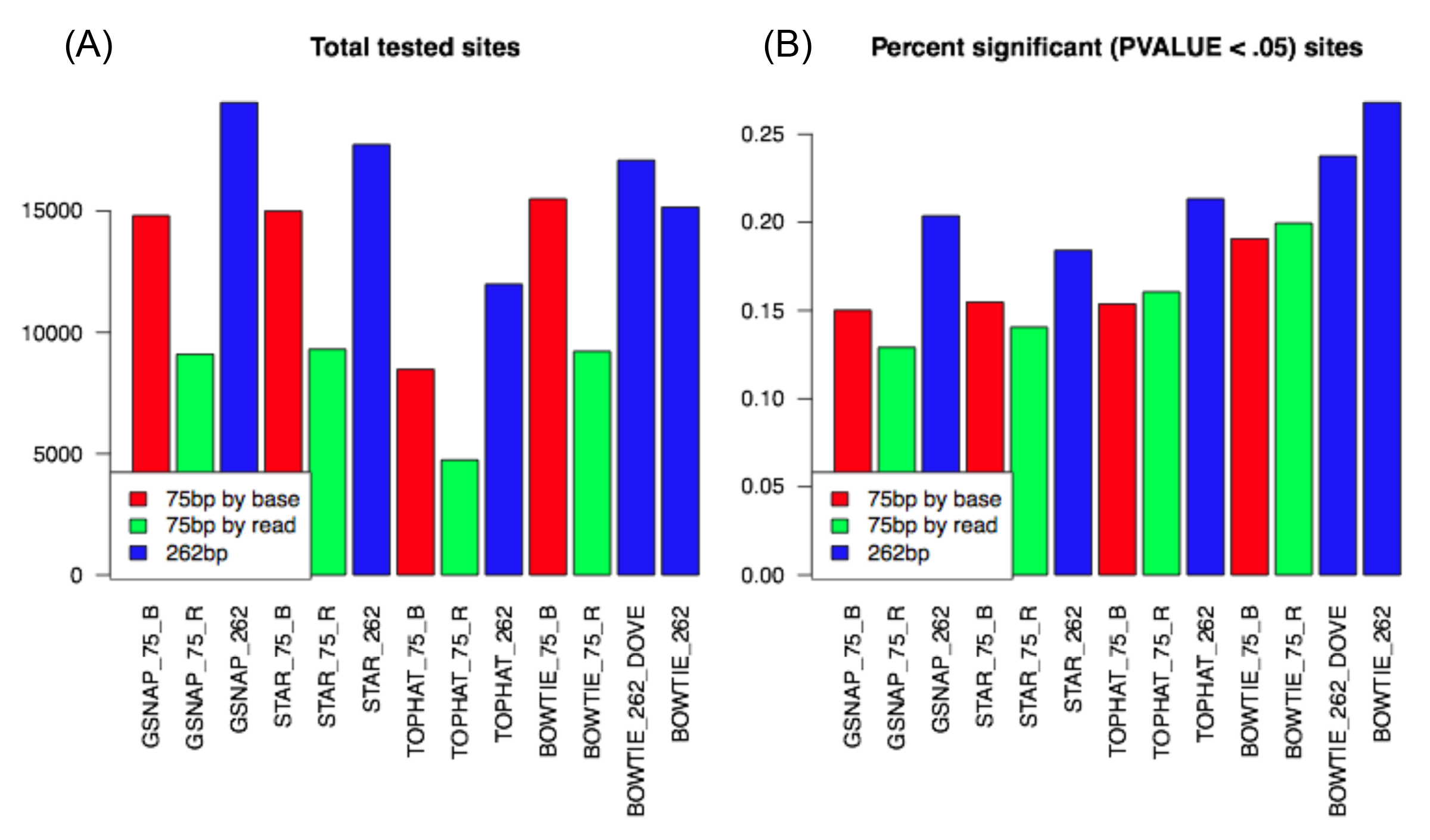

Supplement: Figure S11 — Comparison of read mapping tools and read length for ASE detection. A. Presents the total number of heterozygous sites tested per mapper (Bowtie, STAR, GSNAP, and Tophat) and read length (75 bp and 262 bp) combination. The 75 bp read length data was subsampled to mirror the number of bases (red) and reads (green) in the 262 bp data. The number of tested sites is very similar between STAR and GSNAP regardless of read length, while the number of tested sites for Tophat and Bowtie is significantly lower (on the order of a few thousand sites). This result is in keeping with the number of mapped reads from STAR and GSNAP versus Bowtie and Tophat. B. Percent of significant ASE sites (pvalue ¡.05) for each mapper and read length pair. In general, the long read samples, regardless of mapper, showed a higher percentage of significant sites. L75 was randomly subsampled down to the same number of bases (L75B) and the same number of reads (L75R) as L262. (TIF) [file pone.0108095.s011.tif]

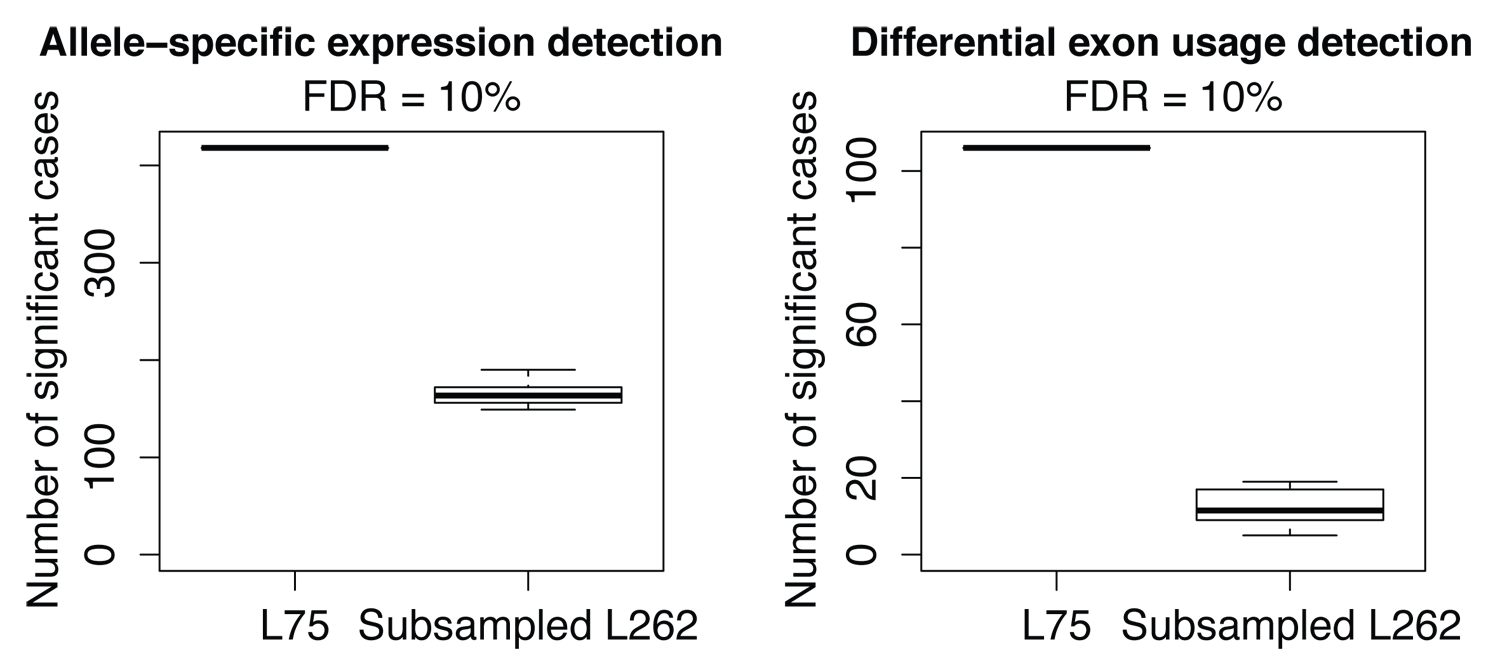

Supplement: Figure S12 — Comparison of allele-specific pattern detection with matching monetary costs. L262 was subsampled down to 45.45% of the original data to match its monetary cost with that of L75. This severely reduces the amount of information contained in the subsampled L262 library, and thus results in a significantly fewer discoveries compared to L75. (TIF) [file pone.0108095.s012.tif]

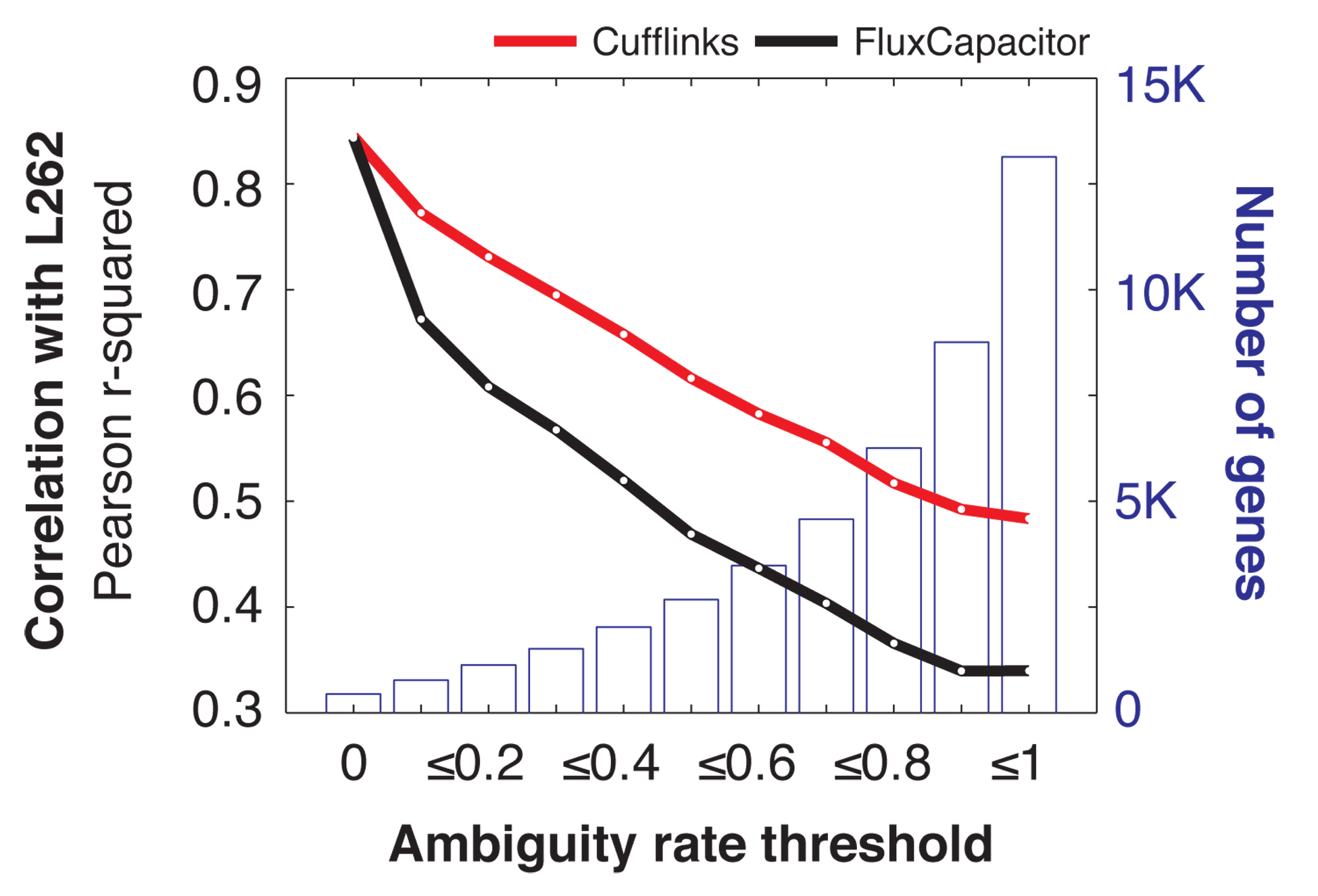

Supplement: Figure S13 — Comparison of Cufflinks and FluxCapacitor based on varying degrees of ambiguity. We calculated the ambiguity rate for each gene as the number of reads mapped to multiple isoforms divided by the number of total reads mapping to that gene in L262. Then, we compared how well transcript abundance estimates from Cufflinks and FluxCapacitor derived from L75 data correlate with the transcript abundances measured directly by L262 unambiguous reads for the subset of genes with multiple isoforms that have at least ten nucleotides uniquely assigned to any single transcript and have ambiguity rates below a certain threshold. This threshold is varied between zero and one to obtain the curves in the figure. We expect abundance estimates based on unambiguous reads in L262, which we used as the ground truth, to be more accurate for genes with lower ambiguity rates. (TIF) [file pone.0108095.s013.tif]
